# Successful Hematopoietic Stem Cell Transplantation Following a Cyclophosphamide-Containing Preparative Regimen with Concomitant Phenobarbital Administration

**DOI:** 10.1155/2012/721857

**Published:** 2012-10-11

**Authors:** Catherine Weber, Heather Kasberg, Edward Copelan

**Affiliations:** ^1^Department of Pharmacy, Cleveland Clinic, 9500 Euclid Avenue, Cleveland, OH 44195, USA; ^2^Department of Bone Marrow Transplantation, Taussig Cancer Institute, Cleveland Clinic, 9500 Euclid Avenue, Cleveland, OH 44195, USA; ^3^Department of Hematologic Oncology and Blood Disorders, Taussig Cancer Institute, Cleveland Clinic, 9500 Euclid Avenue, Cleveland, OH 44195, USA

## Abstract

Cyclophosphamide is an immunosuppressive agent and an anticancer prodrug which requires bioactivation catalyzed primarily by cytochrome P450 enzymes in order to be transformed into its active alkylating compounds. Concomitant administration of drugs known to inhibit or induce this enzyme system is a clinical concern. Herein, we present the case of a chronically ill 21-year-old patient who received high-dose cyclophosphamide, equine antithymocyte globulin (eATG), and total body irradiation (TBI) followed by an allogeneic hematopoietic stem cell transplant (HSCT) for severe aplastic anemia. Throughout her hospitalization, she continued to receive quadruple anticonvulsant therapy including phenobarbital for her long-standing seizure history. The preparative regimen was tolerated well aside from a hypersensitivity reaction to eATG, and minimal cyclophosphamide-related toxicities. Safe and effective administration of high-dose cyclophosphamide was possible with multidisciplinary care consisting of physician, nursing, pharmacy, neurology consultation, as well as social work and case management.

## 1. Introduction

Allogeneic hematopoietic stem cell transplantation is curative in most patients with aplastic anemia. This procedure is associated with toxicities from the preparative regimen and subsequent immunosuppressive therapy, as well as graft versus-host disease (GVHD) and graft failure. Cyclophosphamide is a standard component of preparative regimens for allotransplantation; however, the parent drug requires metabolism primarily by cytochrome P450 enzymes in order to produce its active alkylating compounds, and concomitant administration of interacting medications is of particular concern in the setting of HSCT due to the intensity of the preparative regimen administered.

The activity of the relevant metabolic pathways is affected by a variety of drugs, including phenobarbital, an inducer of P450B and P450A enzymes [1]. Exposure to the active metabolites of cyclophosphamide is increased in the presence of concomitant phenobarbital administration. Although concern for a potential drug-drug interaction exists, the details surrounding this interaction have not yet been fully elucidated and to our knowledge, the only available literature published to date has not been among human subjects [1–6].

## 2. Case Presentation

A 19-year-old woman was in excellent health until she developed viral encephalitis and a prolonged coma requiring tracheostomy, percutaneous endoscopic gastrostomy (PEG) tube placement, and a long course of rehabilitation. The encephalopathy was complicated by chronic, refractory seizures occurring 3-4 times per week, each lasting for 30 seconds to 1.5 minutes. The character of the seizures varied; at times, they were tonic-clonic and at other times, absence seizures. She was managed with multiple anticonvulsants including: phenobarbital, levetiracetam, lacosamide, lamotrigine, and felbamate. She had reduced long- and short-term memory, reduced functional status, poor high level rationalization, generalized muscle weakness requiring use of a walker, and long-standing urinary incontinence and was improving while receiving 24-hour care in a skilled nursing facility. 

Within six months of beginning anticonvulsant therapy, pancytopenia was noted on routine blood work. Bone marrow biopsy revealed a markedly hypocellular marrow indicative of aplastic anemia. She was treated with intravenous immunoglobulin (IVIG) for six months and then received equine antithymocyte globulin (eATG) and cyclosporine without any improvement in blood counts. She was unable to achieve therapeutic cyclosporine levels, which was felt to be a result of interactions with anticonvulsants. She developed hypersensitivity reactions to eATG. The patient required multiple hospitalizations for bacteremia and soft tissue infections and became progressively more transfusion dependent over a year and a half following her diagnosis of aplastic anemia, requiring red cell and platelet transfusions weekly.

This cytomegalovirus (CMV)-positive patient was HLA typed and did not have HLA-matched sibling donor, but an 8/8 matched unrelated, female, CMV-positive adult donor was identified. She was 21-years-old when she was admitted to the inpatient bone marrow transplant unit to begin a preparative regimen of intravenous cyclophosphamide (50 mg/kg daily for 4 days), eATG (30 mg/kg daily for 3 days), and TBI (200 cGY once). The pretransplant preparative regimen is outlined in Table 1. Anticonvulsant medications continued throughout the preparative regimen included: lacosamide 300 mg by mouth twice daily, lamotrigine 400 mg by mouth twice daily, levetiracetam 2,000 mg by mouth twice daily, and phenobarbital 800 mg by mouth daily given in divided doses every 8 hours. The preparative regimen was tolerated well with the exception of hypersensitivity reactions during infusions of eATG consisting of high-grade fevers, chills, rigors, tachycardia, hypotension, low oxygen saturations, and an increased number and length of absence seizures. The second and third doses of eATG were infused over eight hours with a slow increase in infusion rate as the patient tolerated the infusion. 

Donor bone marrow with a total nucleated cell (TNC) count of 4.89 × 10^8^/kg and CD34 count of 3.13 × 10^6^/kg was infused. Graft-versus-host disease prophylaxis consisted of standard dose methotrexate and tacrolimus. The patient maintained therapeutic tacrolimus levels while receiving 2 mg daily via intravenous continuous infusion, with levels ranging between 5.1 and 17 ng/mL. She developed minimal preparative regimen-related toxicities aside from mild esophagitis and nausea. She was able to eat, drink, walk, and take oral medications throughout the hospitalization. Due to increased somnolence, a phenobarbital level was obtained on day +20 and was 91.5 *μ*g/mL. Electroencephalography (EEG) monitoring was negative at that time, none of the antiepileptic drugs or doses were adjusted per the direction of her neurologist, and her seizures continued to remain at baseline. Neutrophil engraftment occurred on day +20, the patient was discharged on day +23, and platelet engraftment occurred by day +26.

On day +26, she was noted to have generalized erythema of her neck, back, and anterior chest wall. Skin biopsy was positive for acute GVHD, and she began treatment with prednisone at 1 mg/kg. The rash resolved completely after two weeks, and a prednisone taper was begun. At day +100 she had no evidence of acute GVHD. She developed sub-therapeutic levels of tacrolimus after converting to the oral formulation, and the dose was continually increased.  Table 2 depicts our patient's tacrolimus trough levels with respect to dose. She has not required red blood cell or platelet transfusion since day +13 and day +18, respectively, and is now out four months from her transplant. Complete donor chimerism was obtained on day +28 and has been maintained since that time. She continues to be followed by her neurologist, who managed the antiepileptic medications and monitored her seizures during the hospitalization.

## 3. Discussion

**Figure 1:**
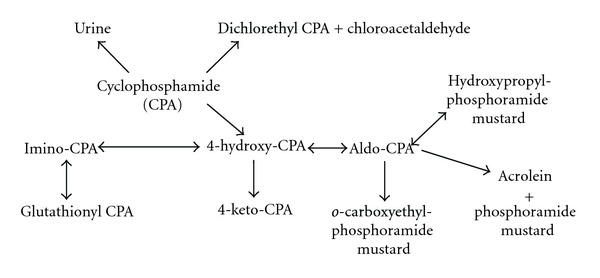
Cyclophosphamide metabolism.

Cyclophosphamide is an anticancer prodrug which requires metabolic biotransformation by a 4-hydroxylation reaction catalyzed primarily by cytochrome P4502B and P4502C enzymes in order to produce its active alkylating compounds, 4-hydroxy-cyclophosphamide, and aldophosphamide [1]. An alternative metabolic pathway for the parent compound involves *N*-dechloroethylation which is catalyzed by P4503A, yielding dechloroethyl-cyclophosphamide and chloroacetaldehyde, an inactive compound, and one associated with unwanted toxicities including neurotoxicity and urinary tract toxicity, respectively. *N*-dechloroethylation is thought to be the minor metabolic pathway for cyclophosphamide, but notable interpatient variability exists [7–9]. The metabolism of cyclophosphamide is displayed in Figure 1.

Substantial basic work has helped to clarify not only the metabolic pathways of cyclophosphamide, but also how these metabolites relate to its toxicity and efficacy [10]. Exposure to the metabolite *o*-carboxyethyl-phosphoramide mustard has been shown to correspond to toxicities including sinusoidal obstructive syndrome and hyperbilirubinemia, and to negatively impact nonrelapse mortality and survival. These results confirm that increased 4-hydroxylation of cyclophosphamide leads to more toxicity and worse outcomes. 

Phenobarbital is an anticonvulsant which is an inducer of P450A, P450B, and P450C enzymes, with the potential to increase the exposure to the active metabolites of cyclophosphamide if administered concomitantly [1, 6, 11]. To our knowledge, the only available literature published to date regarding this particular interaction has been based on animal models [1–3, 5, 6]. Early evidence of an interaction between these two agents was first evaluated in rats, where it was found that phenobarbital-treated rats were exposed to less parent drug and to a higher peak of alkylating metabolites [3]. However, due to an apparent shorter serum half-life of both cyclophosphamide and its metabolites, a similar area under the curve of the alkylating metabolites was documented. Observing shorter survival of phenobarbital-treated mice provided additional evidence albeit in the animal model that this drug interaction may decrease the antitumor activity of cyclophosphamide [2]. 

A cultured human hepatocyte model has also been used to investigate whether hepatic cyclophosphamide and ifosfamide 4-hydroxylation reactions are subject to modulators of the cytochrome P450 enzyme system [6]. Phenobarbital was shown to increase the activation of cyclophosphamide by at least 2-3-fold; ifosfamide activation was increased by approximately 4-fold. This model also demonstrated that cyclophosphamide may induce its own 4-hydroxylation activity, but this result was variable among hepatocyte cultures [6, 12, 13]. Limited information regarding the drug-drug interaction between ifosfamide and cytochrome P450-inducing agents in human subjects exists [14–16]. Although pharmacokinetic parameters are affected, no difference was found with respect to area under the curve of the active metabolites [15, 16]. 

After thorough review of the literature and consideration of our complex patient, it was decided to administer the full, standard dose of cyclophosphamide. Dose modification was not felt to be necessary due to the anticipated unchanged cumulative exposure to the alkylating metabolites of cyclophosphamide when phenobarbital is administered concomitantly. Although overexposure to certain metabolites corresponds to worse outcomes, until further supportive in vivo clinical data of the phenobarbital-cyclophosphamide interaction are published, we believe that administering an unadjusted dose was the most appropriate management. Our patient tolerated this preparative regimen well, obtained complete donor chimerism within 30 days with good blood counts, and continues to improve clinically. Although no doubt a drug-drug interaction exists between cyclophosphamide and phenobarbital, substantial interpatient variability also exists regarding cyclophosphamide metabolism and its own autoinduction even in the absence of concomitant drug administration [10]. Until pharmacokinetic drug monitoring of cyclophosphamide is readily available, we encourage thoughtful evaluation and management of all potential drug-drug interactions, conscious foresight of drug-related toxicities, as well as consideration of patient-specific variables prior to the administration of high-dose cytotoxic chemotherapy, and full, multidisciplinary care of these medically complex patients.

## Figures and Tables

**Table 1:** Cyclophosphamide/TBI/eATG Chemotherapy.

Day −5	Cyclophosphamide 50 mg/kg IV

Day −4	Cyclophosphamide 50 mg/kg IV
	Equine antithymocyte globulin 30 mg/kg IV

Day −3	Cyclophosphamide 50 mg/kg IV
	Equine antithymocyte globulin 30 mg/kg IV

Day −2	Cyclophosphamide 50 mg/kg IV
	Equine antithymocyte globulin 30 mg/kg IV

Day −1	Total body irradiation 200 cGY

Day 0	Marrow infusion

Note: all doses were given based on adjusted body weight.

**Table 2:** Tacrolimus trough levels by dose.

Days after HSCT	Trough level (ng/mL)	Tacrolimus dose
Day +1	10.8	2.6 mg IVCI
Day +5	17.0	2.6 mg IVCI
Day +8	12.7	2 mg IVCI
Day +12	8.3	2 mg IVCI
Day +15	12.1	2 mg IVCI
Day +19	10.4	2 mg IVCI
Day +22	5.1	2 mg PO q12h
Day +26	2.9	2.5 mg PO q12 h
Day +33	3.1	2.5 mg PO q12 h
Day +40	3.1	2.5 mg PO q12 h
Day +47	4.1	2.5 mg PO q12 h
Day +54	3.5	3 mg PO qAM; 2.5 mg PO qPM
Day +57	3.9	3 mg PO qAM; 2.5 mg PO qPM
Day +61	3.5	3 mg PO qAM; 2.5 mg PO qPM
Day +68	4.5	3 mg PO q12 h
Day +77	6.0	3 mg PO q12 h
Day +85	6.4	3 mg PO q12 h
Day +96	4.2	3 mg PO q12 h
Day +116	4.0	3 mg PO q12 h
